# Do Children With Developmental Language Disorder Activate Scene Knowledge to Guide Visual Attention? Effect of Object-Scene Inconsistencies on Gaze Allocation

**DOI:** 10.3389/fpsyg.2021.796459

**Published:** 2022-01-07

**Authors:** Andrea Helo, Ernesto Guerra, Carmen Julia Coloma, Paulina Aravena-Bravo, Pia Rämä

**Affiliations:** ^1^Departamento de Fonoaudiología, Facultad de Medicina, Universidad de Chile, Santiago, Chile; ^2^Departamento de Neurociencias, Facultad de Medicina, Universidad de Chile, Santiago, Chile; ^3^Centro de Investigación Avanzada en Educación, Instituto de Educación—IE, Universidad de Chile, Santiago, Chile; ^4^Escuela de Psicología, Pontificia Universidad Católica de Chile, Santiago, Chile; ^5^Integrative Neuroscience and Cognition Center (UMR 8002), CNRS, Université Paris Descartes, Paris, France

**Keywords:** scene knowledge, object-scene inconsistencies, Developmental Language Disorder, visual scene, eye-movements

## Abstract

Our visual environment is highly predictable in terms of where and in which locations objects can be found. Based on visual experience, children extract rules about visual scene configurations, allowing them to generate scene knowledge. Similarly, children extract the linguistic rules from relatively predictable linguistic contexts. It has been proposed that the capacity of extracting rules from both domains might share some underlying cognitive mechanisms. In the present study, we investigated the link between language and scene knowledge development. To do so, we assessed whether preschool children (age range = 5;4–6;6) with Developmental Language Disorder (DLD), who present several difficulties in the linguistic domain, are equally attracted to object-scene inconsistencies in a visual free-viewing task in comparison with age-matched children with Typical Language Development (TLD). All children explored visual scenes containing semantic (e.g., soap on a breakfast table), syntactic (e.g., bread on the chair back), or both inconsistencies (e.g., soap on the chair back). Since scene knowledge interacts with image properties (i.e., saliency) to guide gaze allocation during visual exploration from the early stages of development, we also included the objects’ saliency rank in the analysis. The results showed that children with DLD were less attracted to semantic and syntactic inconsistencies than children with TLD. In addition, saliency modulated syntactic effect only in the group of children with TLD. Our findings indicate that children with DLD do not activate scene knowledge to guide visual attention as efficiently as children with TLD, especially at the syntactic level, suggesting a link between scene knowledge and language development.

## Introduction

Our surrounding visual environment provides a rich and predictable context with typical configurations. First, certain objects (e.g., a saucepan) are more likely to appear in certain contexts (e.g., kitchen). Second, objects are more likely to be located in certain places within the scene (e.g., the saucepan usually rests on the stove). Through visual experience, scene-based rules are stored in the long-term memory generating scene knowledge (Potter, 1975; Mandler and Johnson, 1976; Hock et al., 1978; Bartlett, 1995). This knowledge allows viewers to extract the meaning of a visual scene rapidly and to generate expectations about object-scene *what* and *where* relations. This, in turn, facilitates objects’ identification and reduces the cognitive demand of scene processing (Draschkow and Võ, 2017; Võ et al., 2019). Inspired by the linguistic domain, these two configurations have also been described as semantic and syntactic relations (Biederman et al., 1982; Võ and Henderson, 2009, 2011; Võ and Wolfe, 2013; Võ et al., 2019). Semantic relations refer to taxonomic and functional links between objects, and their probability to belong to certain contexts (e.g., a saucepan in a kitchen). In turn, syntactic relations refer to the location of these objects within the structure of the scene (e.g., the saucepan on the stove).

In language, listeners generate semantic representations and extract syntactic rules from relatively predictable linguistic contexts. Likewise, through visual experience, viewers extract semantic object-object relations and object-scene relations, and the syntactic rules from their surrounding visual environments. Based on these similarities, it has been proposed that language and scene knowledge might share some underpinning cognitive mechanisms (Võ et al., 2019; Öhlschläger and Võ, 2020). This suggestion finds support in neurophysiological evidence. Võ and Wolfe (2013) showed that visual semantic and syntactic inconsistencies during scene viewing elicited two different event-related potential (ERP) components. Object-scene semantic inconsistencies elicited an N400, while object-scene syntactic inconsistencies elicited a P600 response. There is extensive literature in the language domain linking the N400 component with semantic processing and the P600 component with syntactic processing (see, Kutas and Federmeier, 2011; Leckey and Federmeier, 2020, for reviews).

According to the cognitive guidance theory (Henderson and Hayes, 2017; Henderson et al., 2018), an internal representation of scenes (i.e., scene knowledge) guides visual attention during scene exploration, and gaze is often directed to regions that are relevant either for scene understanding or for achieving a task goal (Loftus and Mackworth, 1978; De Graef et al., 1990; Einhäuser et al., 2008; Castelhano et al., 2009; Võ and Henderson, 2009, 2011; Mills et al., 2011). In this context, evidence has shown that both, semantics (e.g., a sock in the kitchen; De Graef et al., 1990; Henderson et al., 1999; Võ and Henderson, 2009) and syntactic (e.g., a saucepan on the floor; De Graef et al., 1990; Võ and Henderson, 2009; Öhlschläger and Võ, 2017; Võ et al., 2019) scene-object inconsistencies strongly influence gaze allocation, attracting the gaze of observers and increasing the number of fixation landings and looking times. In addition to scene knowledge, low-level visual features of the scene, such as saliency, play an essential role in guiding visual attention. Saliency can be defined as the difference between perceptual properties (i.e., color, intensity, contrast, and edge orientation) of a visual stimulus compared to the near visual input (Koch and Ullman, 1985; Itti and Koch, 2000; Le Meur et al., 2006). The more salient the regions are, the more likely they are to be fixated (Itti and Koch, 2001; Treue, 2003). Therefore, visual attention is influenced by the interaction between cognitive mechanisms associated with scene knowledge and the perceptual features of images.

Developmental studies have shown that both scene knowledge (Helo et al., 2017; Öhlschläger and Võ, 2020) and perceptual features (Açık et al., 2010; Helo et al., 2014, 2017) modulate visual attention in young children. Helo et al. (2017) examined the interaction between perceptual features and scene knowledge in toddlers, showing that semantic object-scene inconsistency effects appeared in 2-year-olds, but only for highly salient objects. More recently, Öhlschläger and Võ (2020) examined scene-knowledge guidance in children between 2 and 4 years old by measuring looking time to scene-object inconsistencies. They showed that inconsistency effects were observable in 4-year-olds (but not in younger children). Taken together, these findings suggest that scene knowledge is available to guide visual attention by age four. Although it might emerge before this age, scene knowledge gaze guidance seems to rely on the presence of additional clues, such as saliency.

The same authors showed an interaction between language skills and scene knowledge for visual guidance during scene exploration (Helo et al., 2017; Öhlschläger and Võ, 2020). Helo et al. (2017) assessed whether productive skills were related to scene knowledge, showing that while looking time to semantically inconsistent objects was not modulated by the toddler’s expressive vocabulary, children with higher vocabulary were more attracted by consistent objects than children with lower vocabulary. Similarly, Öhlschläger and Võ (2020) studied whether language skills (i.e., concept classification skills) modulated children’s gaze allocation. They observed that children with better language skills exhibited a greater difference between consistent and inconsistent objects. This tendency (marginally significant) was driven by a decrease in the looking time to the consistent objects in children with better language skills. These findings suggest a link between language skills and visual attention guidance during scene perception. Yet, the existing findings are not conclusive, and the relation between language skills and scene knowledge development needs further investigation.

A way to further examine the interaction between scene knowledge and language development is by studying a clinical population with atypical language development. Developmental Language Disorder (DLD) is a condition that affects language acquisition and development (comprehensive and/or expressive) in one or more areas of language, interfering with social and educational everyday life. These difficulties are not due to neurobiological causes such as neurological damage, hearing deficit, cognitive impairment, or environmental deprivation (Bishop et al., 2017). Children with DLD usually show a heterogeneous linguistic profile (Parisse and Maillart, 2009) but one central aspect of this disorder is the difficulty in their grammar abilities both at morphological and syntactic level (van der Lely, 1998; Bedore and Leonard, 2001; Conti-Ramsden et al., 2001; van der Lely et al., 2004; Moscati et al., 2020). In addition, these children often present vocabulary deficits and weaker semantic representations (Kail et al., 1984; Gray et al., 1999; McGregor et al., 2002; Andreu et al., 2012), yet to a lesser extent relative to syntactic difficulties.

It has been proposed that the grammar difficulties observed in children with DLD are due to problems extracting rules from the linguistic context (Ullman et al., 2020). Previous evidence indicates that these difficulties in the extraction of regularities go beyond the linguistic domain (see Obeid et al., 2016). If these difficulties also affect scene knowledge guidance during scene exploration, it might suggest a common underlying cognitive mechanism for scene knowledge and language development.

The current study examined scene knowledge in a group of children with and without DLD. Specifically, we assessed visual attention and gaze allocation to syntactic, semantic, and semantic-syntactic object-scene inconsistencies in these two groups of children with different linguistic profiles. Since perceptual features also have a strong effect on gaze allocation, we include objects’ saliency as a predictor of the object-scene inconsistency effect. We propose that if semantic and syntactic processing in the visual and language domain share some underlying cognitive mechanisms, children with DLD would show difficulties in object-scene violation detection, particularly at the syntactic level since grammar is a hallmark of this disorder.

## Materials and Methods

### Participants

Our sample consisted of 40 Spanish-speaking monolinguals preschoolers, including 20 children with a diagnosis of DLD (range = 5;4–6;6, mean age = 5;9, 6 girls), and a control group of 20 children with typical language development (henceforth TLD; range = 5;4–6;6, mean age = 6;0, 8 girls). We conducted the study within this age range because the DLD diagnosis can be fully confirmed only from 5 years of age (Aguado et al., 2015). Participants assisted to the last preschool year at Chilean public schools that had implemented a Government Program Service for children with DLD (Integration Program or *Programa de Integración Escolar*, PIE).^1^ Children in the TLD group were classmate of the children with DLD paired by age. Parents signed an informed consent form, while all children verbally agreed to participate. All experiments and procedures were approved by the faculty’s Ethics Committee of the University of Chile.

### Sample Selection

All children in the TLD group had been screened by schoolteachers discarding language difficulties (or in other developmental domains), as part of a standard procedure in schools with PIE at the beginning of preschool. Additionally, we asked the head teachers to identify children with no history of language difficulties, and with a normal school performance. Finally, we also assessed vocabulary skills through the Expressive Vocabulary subtest of the CELF-4 in this group and all recruited children scored within the average range (i.e., scaled score at or above –1SD below the mean).

Children with DLD were diagnosed by a multidisciplinary team led by the speech therapists at their school based on standard guidelines dictated by the Chilean Ministry of Education ( Decree Law No. 170, 2010 ). These guidelines follow the same criteria for clinical diagnoses as stated in the Diagnostic and Statistical Manual of Mental Disorders [American Psychiatric Association (APA), 2013] and the International Statistical Classification of Diseases and Related Health Problems [World Health Organization (WHO), 2019].

The DLD diagnosis, made by the speech therapist, is based on the battery of tests indicated by the Ministry of Education, including the Test for the Evaluation of Phonological Simplification Processes (TEPROSIF-R; Pavez et al., 2008; Cronbach’s α = 0.90) and the Allen Toronto’s Exploratory Test of Spanish Grammar (Pavez, 2003) that assesses grammatical performance through an expressive (Cronbach’s α = 0.77) and a receptive (Cronbach’s α = 0.83) subtest. Children might have different profiles based on their performance in these tests. However, the presence of grammar difficulties is required for a DLD diagnosis. Thus, all children with DLD in our study had scored within the deficit level in the grammar dimension either at expressive or receptive subsets in The Toronto’s Exploratory Test of Spanish Grammar (two standard deviations under the Chilean norms). The Toronto’s Exploratory Test of Spanish Grammar has proved to differentiate children with DLD from children with TLD based on grammatical performance in a Chilean sample (Pavez, 2003). Additionally, according to the Chilean Ministry of Education guidelines, a medical, pedagogical, and psycho-pedagogical evaluation must be conducted to dismiss any other disorders that might affect language development.

Moreover, our research team used the CELF-4 (Semel et al., 2003) to further assessed children with DLD through an internationally accepted battery. This evaluation made by a speech therapist from our research team included four subtests of the CELF-4 assessing grammar and lexical-semantic skills. We applied the Formulated Sentences and the Word Structure subtest to assesses grammar skills, and the Expressive Vocabulary, the Receptive Word Classes, and Expressive Word Classes to assess lexical semantic skills (see Table 1). We used the CELF-4 norms for the Hispanic population of the United States due to the lack of Chilean norms. These norms are widely used by researchers in Spanish-speaking countries (see, e.g., Acosta et al., 2013; Ramírez-Santana et al., 2019; Sepulveda et al., 2021). Those children who fell under 1.25 standard deviation from the mean in a given subtest were classified as having low performance in that subtest. All children with DLD had deficits at the grammar level (that is, a score under 1.25 SDs on the Formulated Sentences, the Word Structure, or both). From those, five children showed low performance only at grammar level and 15 children had low performance at the grammar and the semantic level (see Supplementary Material for more detail). None showed low performance at expressive vocabulary subtests. However, they differed significantly in the scores with the control group (TLD mean raw score = 29.3, SD = 8.8; DLD mean raw score = 16.45, SD = 4.34; Welch two sample *t*-test, *t* = −5.92, *p* < 0.001).

**TABLE 1:** Scaled scores in evaluates CELF-4 subtests for children with DLD.

CELF-subtests	Mean scalar scores	Range	Mean percentile	Range
Formulated sentences subtest	4.4 (1.50 SD)	3–9	5	1–25
Word structure subtests	6.38 (2.48 SD)	2–10	17	0.4–50
CELF–expressive vocabulary	10.1 (1.12 SD)	8–12	51	37–75
Receptive word classes subtests	5.64 (3.53 SD)	3–12	16	0.1–75
Expressive word classes subtests	5.92 (1.72 SD)	3–12	12	1–50

Children in both groups had normal hearing, measured by screening for hearing impairment (at or below 20 dB; CDC, 2020) and had normal non-verbal cognitive skills assessed by Raven’s colored progressive matrices test (≥percentile 25).

### Material and Design

**FIGURE 1:**
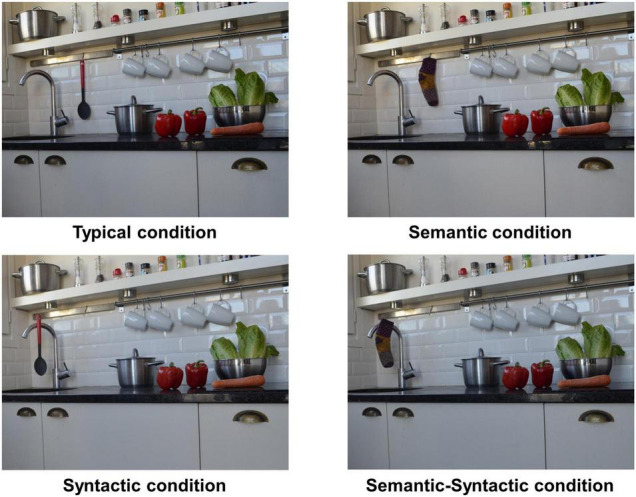
Example of a every experimental condition for a single scene.

We produced 20 visual scenes depicting four types of real-life everyday indoor scenes (i.e., five bathrooms, five kitchens, five living rooms, and five bedrooms) using a Nikon D5100 camera with a resolution of 1024 × 768 pixels. Each scene contained a target object in a different object-scene relation, generating four versions, one for each experimental condition or trial type (i.e., typical, semantic, syntactic, and semantic-syntactic trials; see Figure 1). In the *typical* trials, the target was consistent with the scene (e.g., a ladle hanging from a kitchen’s hook), in the *semantic* trials the scene included a target that did not belong to the scene context (e.g., a sock hanging from a kitchen’s hook), in the *syntactic* trials the target belonged to the scene but was placed in a wrong position (e.g., a ladle hanging from a kitchen’s water tap) and in the *semantic-syntactic* trials the target was semantically inconsistent with the scene context and was placed in a wrong position (e.g., a sock hanging from a kitchen’s water tap). Besides target objects (typical, semantic, syntactic, or semantic-syntactic), each scene included a control object always semantically and syntactically consistent with the scene. This object appeared as a semantic inconsistent target object in another scene and was included to control for the interest that a particular object might elicit on its own. An area of interest (AOI) was defined for each target and control object. There were no differences in the AOIs size between conditions. The saliency of target and control objects was ranked from 1 to 15 (1 being the most salient) using the MATLAB Saliency Toolbox (Walther and Koch, 2006). This toolbox creates a saliency map that allows estimating the saliency level of each region in an image. Repeated measures analyses of variance showed no differences in saliency between conditions, *F* < 1. Using a Latin square design, we created four experimental lists that rotated the object-scene experimental condition for each scene. Thus, every participant saw each scene in only one condition, and the same number of conditions, items, and scene type across the experiment. The position of each type of object was counterbalanced across items between the four quadrants of the scene.

### Procedure

Participants were seated in front of the computer screen in an isolated room at their school. They were invited to explore the scenes freely. Before the experiment started, a 5-point calibration was implemented. On each trial, a central fixation point was initially presented, after which a visual display appeared on the screen for 7 s. Participants’ eye movements were recorded during the whole experiment, which lasted approximately 5 min.

### Apparatus

Eye movements were sampled monocularly at 500 Hz using the remote mode of a Desktop EyeLink 1000 Plus eye-tracker (SR Research). Pictures were displayed using a 24-inch high-precision display (BenQ XL2430) at 1024 × 768 pixels placed approximately at 60 cm from the participant.

### Data Analysis

We produced four distinctive dependent variables using the Data Viewer software (SR Research). The first three were foveal measures indicating the degree of attention allocated at distinctive time scales. These measures (listed from latest to earliest processing time) included dwell time proportion (i.e., looking time in the AOI divided by the total looking time of the trial; see Öhlschläger and Võ, 2020), first-pass gaze duration (i.e., looking time in the AOI from the first time participants’ gaze enters the AOI until they leave this region; see Öhlschläger and Võ, 2020), and first fixation duration (i.e., duration of the first fixation to the AOI; see De Graef et al., 1990; Henderson et al., 1999). The fourth measure (and the earliest) was an extrafoveal measure (i.e., first saccade start time), reflecting the first moment in which an object attracted participants’ attention (see Võ and Henderson, 2009). Finally, we calculated the percentage of total looking time to the scene by group to measure engagement in scene exploration.

Inferential analysis was carried out within the framework of linear mixed modeling (see Baayen et al., 2008), implemented with the R software (R Core Team, 2021) using the *lme4* (for linear regression models, LMER) and *glmmTMB* (for generalized regression models, GLM) packages (Bates et al., 2015; Brooks et al., 2017). We estimated *p*-values using the *lmerTest* package (Kuznetsova et al., 2017). Each model included the main effects of the saliency (as a scaled continuous predictor), children’s group (DLD vs. TLD), and experimental condition (i.e., syntactic, semantic, or semantic-syntactic violation, or control, vs. typical trials), as well as the interaction between the experimental condition, saliency, and group.

We implemented two versions of the same regression model to evaluate the effect of experimental conditions and saliency on each group of children. We rotated each group as the intercept via a treatment contrast (see Schad et al., 2020). Thus, for each dependent variable, we reported two regression models. Following Barr et al. (2013) we pursued maximal models for each regression; each model had a random intercept for participants, with random slopes for the main effect of each experimental condition and saliency, and a random intercept for items, with the same random slopes plus the main effect of the group. First-pass dwell time and first fixation duration were log-transformed, prior data trimming (fixations <80 ms and >1,000 ms) of the latter. Significant effects are reported within the text, and full model results can be found in the Supplementary Material.

## Results

Both groups of children evidenced a high percentage of trial total looking time (DLD: mean = 0.807, SD = 0.168; TLD: mean = 0.823, SD = 0.135), reflecting that children from both groups were engaged in the exploration of the scenes and that there were no significant differences between the groups (McNemar’s χ^2^ = 0.5, *df* = 1, *p* = 0.5).

### Proportion of Total Looking Time to the Area of Interest

**FIGURE 2:**
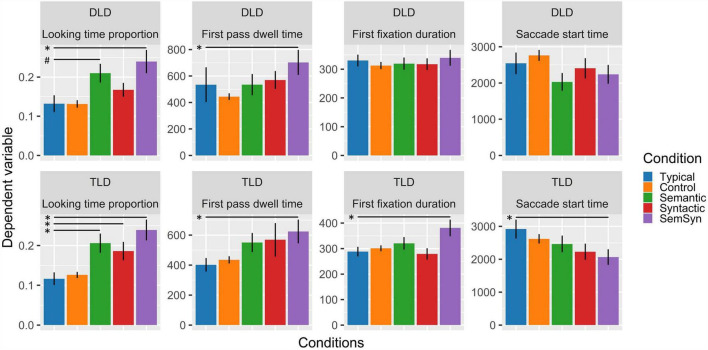
Mean response per group, experimental condition, and measure. Error bars represent within-subject design standard error of the mean (SE). Stars (*) mark significant main effects (*p* < 0.05) and pounds (#) mark marginally significant main effects (*p* < 0.1).

**FIGURE 3:**
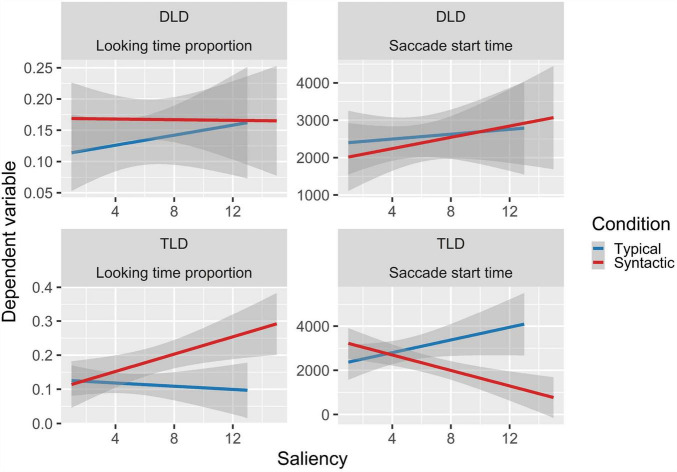
Linear relations between saliency and the two dependent variables where a significant interaction effect was observed. Shaded areas represent the standard error of the mean (SE).

In the DLD group the GLM showed a significantly higher proportion of looks to the semantic-syntactic trials object compared to the typical trials object (β = 0.568, *se* = 0.202, *z* = 2.810, *p* = 0.005), and a marginally significant visual preference for the semantic condition object (β = 0.345, *se* = 0.192, *z* = 1.792, *p* = 0.073). By contrast, the TLD group showed a significantly higher proportion to syntactic (β = 0.417, *se* = 0.183, *z* = 2.280, *p* = 0.023), the semantic (β = 0.513, *se* = 0.192, *z* = 2.676, *p* = 0.007), and semantic-syntactic trials object (β = 0.929, *se* = 0.206, *z* = 4.501, *p* < 0.001) relative to the typical trials object (see Figure 2). In addition, the TLD group exhibit an interaction between the syntactic effect and saliency (β = 0.341, *se* = 0.153, *z* = 2.231, *p* = 0.026), showing an increase in the proportion of looking time to the syntactic (but not the typical) trials object, with decreasing saliency (see Figure 3). Finally, we observed an interaction between group, the syntactic effect and saliency (β = 0.435, *se* = 0.187, *z* = 2.323, *p* = 0.020), mainly driven by the two-way interaction observed in the TLD group, which was absent in the DLD group (see Figure 3).

### First Pass Dwell Time

The LMER analysis showed for both groups only significantly longer looking time to the semantic-syntactic trials object (DLD: β = 0.351, *se* = 0.169, *t* = 2.079, *p* = 0.041; TLD: β = 0.342, *se* = 0.164, *t* = 2.080, *p* = 0.041) relative to the typical trials object in this measure (see Figure 2).

### First Fixation Duration

The TLD LMER model showed only significantly longer looking time to the semantic-syntactic trials object (β = 0.209, *se* = 0.104, *t* = 2.002, *p* = 0.049) compared to the typical trials object in this measure (see Figure 2). The DLD LMER model showed no significant effects.

### Saccade Start Time

The TLD LMER model showed significantly earlier saccades to the semantic-syntactic trials object (β = −766.1, *se* = 374, *t* = −2.046, *p* = 0.045) compared to the typical trials object, and a two-way interaction between the syntactic effect and saliency (β = −1059.6, *se* = 306, *t* = −3.4520, *p* = 0.001), reflecting later saccades to more salient objects in syntactic trials. In addition, both LMER models showed a reliable interaction effect between group, syntactic trials, and saliency (β = −1115.4, *se* = 401, *t* = −2.776, *p* = 0.006). This interaction is also driven by the two-way interaction observed in the TLD group, absent in the DLD group (see Figure 3).

## Discussion

In the present study, we investigated the activation of scene knowledge to guide scene exploration in preschoolers with different linguistic profiles. To do so, we measured looking times to objects that violated the scene-object configuration either semantically, syntactically, or syntactically and semantically in a group of children with DLD and a group of children with TLD. We also introduced scene saliency as a continuous measure in the analysis. The group of children with TLD showed an inconsistency effect in total dwell time for every condition, that is, they looked longer to semantic, syntactic, and semantic-syntactic inconsistent objects compared to consistent objects. In turn, the group of children with DLD showed a significant inconsistency effect only for semantic-syntactic violations. These findings suggest that although children with DLD are attracted to strong scene-objects violations they have not yet consolidated the activation of scene knowledge to guide their visual attention to less strong scene object-violations.

Our findings also showed differences in the efficiency of scene knowledge guidance between groups. First, the group of children with DLD presented an inconsistency effect for semantic-syntactic condition from the first pass dwell time measure whereas children with TLD did so already from the first fixation. This finding indicates that children with DLD need more time than their age-control peers to detect scene-object violations. Second, typically developing children clearly showed semantic and syntactic effects in total looking time while children with DLD showed only trend for the detection of semantic inconsistencies and no effect at the syntactic level. The lack of inconsistencies detection at these level in the DLD group might reflect a less consolidated semantic and syntactic scene knowledge guidance in this population. Alternatively, this finding might reflect a less efficient scene exploration in DLD, which in turn, decreases the chance to reach the target object. However, our analysis of the saccade start time revealed no group effect showing that, overall, both groups reached the AOIs at a similar time (see Supplementary Material). Similarly, we found no differences between groups in the percentage of trial total looking time, which suggests similar exploration skills and engagement with the task in both groups. We propose that these results point to an underdeveloped scene knowledge guidance in the group of children with DLD. Previously, implicit measures of syntactic scene knowledge (i.e., eye movements during scene exploration) have been significantly correlated with explicit measures (i.e., asking children to place toy objects in their corresponding dollhouse room, see Öhlschläger and Võ, 2020). Thus, future research using an explicit measure could confirm that the difficulties we observed in children with DLD are related to the development of scene knowledge.

Our results suggest that the most affected aspect of scene knowledge guidance in DLD is related to syntactic scene-object violations, since the preference for the semantic scene-object violation almost reached statistical significance. Syntactic difficulties are a hallmark of the language deficits in children with DLD and it has been argued that these difficulties obey a cognitive deficit associated with the ability to extract the visual and linguistic regularities from the environment (see, Obeid et al., 2016 for a meta-analysis), deficit that may account for their syntactic difficulties (Ullman and Pierpont, 2005; Obeid et al., 2016; Ullman et al., 2020). Accordingly, these difficulties may be also manifested in the extraction of visual scene regularities affecting the configuration of scene syntax in this population. Although not as strongly affected as the syntactic level, children with DLD often present lexical-semantic deficits (e.g., Gray et al., 1999; McGregor et al., 2002). Accordingly, in the present data, children with DLD exhibited only a trend of semantic inconsistencies detection. Thus, the results from this scene perception study mirror the deficit this population exhibits in the language domain.

In children with TLD, we found an interaction between saliency and the syntactic consistency effect. Extrafoveal measures revealed that the most salient objects were fixed later than less salient objects in the syntactic condition. Interestingly, we observed the same direction of the syntactic effect in later stages (i.e., dwell time), where the syntactic effect increased as saliency decreased. These results differ from previous evidence obtained in adults (Parkhurst et al., 2002; Tatler and Vincent, 2008; Castelhano et al., 2009), and children (Helo et al., 2014) showing that saliency has a stronger influence on gaze allocation during earlier stages of scene processing, while cognitive control becomes more relevant at later processing stages. There is certain agreement that cognitive guidance (e.g., scene knowledge) dominates and modulates perceptual guidance (e.g., saliency) during scene exploration (Henderson, 2007; Henderson et al., 2009, 2018). Also, previous research showed an interaction between saliency and scene-object inconsistencies effects for semantic violations in 2-year-old children (but lack of a main semantic effect), suggesting that scene exploration is not yet fully developed at this age, and saliency is needed to guide children to the AOIs facilitating semantic processing (Helo et al., 2017). Our findings in preschool aged children might be reflecting that syntactic knowledge develops later than semantic knowledge (see, Saarnio, 1990) and is less consolidated even in the TLD group. This, in turn, allowed saliency to play a more relevant role in syntactic trials, at least for this group.

Importantly, saliency did not affect gaze allocation in children with DLD. In this regard, existing evidence suggests that this population has difficulties in the visual domain. For instance, studies have shown than children with DLD are slower in visual detection tasks compared to their age-matched controls (Park et al., 2020; Ebert, 2021). Also, it has been shown that children with DLD present less efficient visual attention engagement (Dispaldro et al., 2013; Dispaldro and Corradi, 2015) and difficulties with visual attentional control (Blom and Boerma, 2020). Likewise, evidence shows that children with DLD present poorer visual recall skills in visual memory tasks (Hoffman and Gillam, 2004) and difficulties with visuospatial working memory (Vugs et al., 2014; Blom and Boerma, 2020) compared to their peers. These difficulties might extend to perceptual feature processing during scene viewing, resulting in diminished influence of saliency on visual attention in this population. Alternatively, the lack of syntactic effect in the DLD group might be obscuring a potential interaction with saliency. Yet, no prior studies have examined saliency guidance of visual attention in DLD, and thus, further research is needed to clarify this issue.

Taken together, our results suggest that children with DLD might have less consolidated scene knowledge guidance, particularly at the syntactic level of the scene. This finding appears to be in parallel with the known deficit profile that children with DLD present in language development. In line with some recent proposals on the shared underlying cognitive mechanism for language and visual processing (Võ and Wolfe, 2013; Võ et al., 2019; Öhlschläger and Võ, 2020), our data suggest that there might be a similar process for the extraction of regularities from our environment, both in the linguistic and the visual domains.

## Data Availability Statement

The raw data supporting the conclusions of this article will be made available by the authors, without undue reservation.

## Ethics Statement

The studies involving human participants were reviewed and approved by Comité de Ética, Facultad de Medicina, Universidad de Chile. Written informed consent to participate in this study was provided by the participants’ legal guardian/next of kin.

## Author Contributions

AH and PR developed the study concept and designed the experiment. EG and AH implemented and analyzed the data. PA-B, CJC, and AH collected the data. AH, EG, and PR drafted the manuscript. CJC and PA-B revised the manuscript. All authors interpreted the results and approved its final version for submission.

## Conflict of Interest

The authors declare that the research was conducted in the absence of any commercial or financial relationships that could be construed as a potential conflict of interest.

## Publisher’s Note

All claims expressed in this article are solely those of the authors and do not necessarily represent those of their affiliated organizations, or those of the publisher, the editors and the reviewers. Any product that may be evaluated in this article, or claim that may be made by its manufacturer, is not guaranteed or endorsed by the publisher.

## Footnotes

1 The PIE program promotes the integration between children with special needs and children with typical development. Both groups attend the same classes and follow the same school’s curriculum, yet children with special needs receive additional educational support.
