# Supplementary material for: Do Children With Developmental Language Disorder Activate Scene Knowledge to Guide Visual Attention? Effect of Object-Scene Inconsistencies on Gaze Allocation
Source: Front Psychol. 2022 Jan 7;12:796459. doi: 10.3389/fpsyg.2021.796459 (PMC8776641; doi:10.3389/fpsyg.2021.796459)
Supplement: Supplementary file 1 [file Table_1.docx]

**Supplementary materials**

**Do Children With Developmental Language Disorder Activate Scene Knowledge to Guide Visual Attention? Effect of Object-Scene Inconsistencies on Gaze Allocation**

Andrea Helo^1,2,3*^, Ernesto Guerra^3^, Carmen Julia Coloma^1,3^, Paulina Aravena-Bravo^1,4^ & Pia Rämä^5^

*^1^Departamento de Fonoaudiología, Facultad de Medicina, Universidad de Chile, ^2^Departamento de Neurociencias, Facultad de Medicina, Universidad de Chile, ^3^Centro de Investigación Avanzada en Educación, Instituto de Educación—IE, Universidad de Chile, ^4^Escuela de Psicología, Pontificia Universidad Católica de Chile, ^5^Integrative Neuroscience and Cognition Center (UMR8002), CNRS, Université Paris*

**Table S1.** Regression analysis results

| **Looking time proportion:** |  |  |  |  |  |
| --- | --- | --- | --- | --- | --- |
| **DLD is intercept** | *β* | *se* | *z* | *p* |  |
| (Intercept) | -1.86 | 0.12 | -15.41 | 0.000 | *** |
| Syntactic | 0.29 | 0.17 | 1.64 | 0.101 |  |
| Semantic | 0.35 | 0.19 | 1.79 | 0.073 | # |
| SemSyn | 0.57 | 0.20 | 2.81 | 0.005 | ** |
| Control | -0.12 | 0.14 | -0.86 | 0.389 |  |
| Group effect | -0.22 | 0.16 | -1.40 | 0.160 |  |
| scale(Saliency) | 0.15 | 0.12 | 1.26 | 0.207 |  |
| Syntactic * Group effect | 0.13 | 0.24 | 0.56 | 0.573 |  |
| Semantic * Group effect | 0.17 | 0.26 | 0.64 | 0.524 |  |
| SemSyn * Group effect | 0.36 | 0.27 | 1.33 | 0.183 |  |
| Control * Group effect | 0.23 | 0.18 | 1.27 | 0.205 |  |
| Syntactic * scale(Saliency) | -0.09 | 0.15 | -0.64 | 0.523 |  |
| Semantic * scale(Saliency) | -0.18 | 0.16 | -1.11 | 0.269 |  |
| SemSyn * scale(Saliency) | -0.05 | 0.20 | -0.25 | 0.806 |  |
| Control * scale(Saliency) | -0.20 | 0.13 | -1.54 | 0.125 |  |
| Group effect * scale(Saliency) | -0.24 | 0.15 | -1.62 | 0.106 |  |
| Syntactic * Group effect * scale(Saliency) | 0.44 | 0.19 | 2.32 | 0.020 | * |
| Semantic * Group effect * scale(Saliency) | -0.11 | 0.21 | -0.53 | 0.596 |  |
| SemSyn * Group effect * scale(Saliency) | 0.15 | 0.24 | 0.61 | 0.542 |  |
| Control * Group effect * scale(Saliency) | 0.31 | 0.17 | 1.90 | 0.058 | # |
|  |  |  |  |  |  |
| **Looking time proportion:** |  |  |  |  |  |
| **TLD is intercept** | *β* | *se* | *z* | *p* |  |
| (Intercept) | -2.08 | 0.13 | -16.30 | 0.000 | *** |
| Syntactic | 0.42 | 0.18 | 2.28 | 0.023 | * |
| Semantic | 0.51 | 0.19 | 2.68 | 0.007 | ** |
| SemSyn | 0.93 | 0.21 | 4.50 | 0.000 | *** |
| Control | 0.11 | 0.14 | 0.76 | 0.446 |  |
| Group effect | 0.22 | 0.16 | 1.34 | 0.181 |  |
| scale(Saliency) | -0.09 | 0.13 | -0.68 | 0.499 |  |
| Syntactic * Group effect | -0.13 | 0.24 | -0.56 | 0.576 |  |
| Semantic * Group effect | -0.17 | 0.26 | -0.63 | 0.530 |  |
| SemSyn * Group effect | -0.36 | 0.27 | -1.33 | 0.184 |  |
| Control * Group effect | -0.22 | 0.18 | -1.23 | 0.218 |  |
| Syntactic * scale(Saliency) | 0.34 | 0.15 | 2.23 | 0.026 | * |
| Semantic * scale(Saliency) | -0.29 | 0.17 | -1.72 | 0.085 | # |
| SemSyn * scale(Saliency) | 0.10 | 0.18 | 0.57 | 0.566 |  |
| Control * scale(Saliency) | 0.11 | 0.14 | 0.80 | 0.423 |  |
| Group effect * scale(Saliency) | 0.24 | 0.15 | 1.62 | 0.104 |  |
| Syntactic * Group effect * scale(Saliency) | -0.43 | 0.19 | -2.26 | 0.024 | * |
| Semantic * Group effect * scale(Saliency) | 0.11 | 0.21 | 0.51 | 0.610 |  |
| SemSyn * Group effect * scale(Saliency) | -0.16 | 0.24 | -0.65 | 0.513 |  |
| Control * Group effect * scale(Saliency) | -0.32 | 0.17 | -1.88 | 0.060 | # |
|  |  |  |  |  |  |
| **First pass dwell time:** |  |  |  |  |  |
| **DLD is intercept** | *β* | *se* | *t* | *p* |  |
| (Intercept) | 5.91 | 0.10 | 58.28 | 0.000 | *** |
| Syntactic | 0.15 | 0.15 | 1.03 | 0.304 |  |
| Semantic | 0.03 | 0.15 | 0.17 | 0.865 |  |
| SemSyn | 0.35 | 0.17 | 2.08 | 0.041 | * |
| Control | -0.06 | 0.11 | -0.55 | 0.585 |  |
| Group effect | -0.11 | 0.14 | -0.77 | 0.440 |  |
| scale(Saliency) | 0.09 | 0.09 | 0.97 | 0.335 |  |
| Syntactic * Group effect | -0.02 | 0.20 | -0.12 | 0.907 |  |
| Semantic * Group effect | 0.16 | 0.20 | 0.76 | 0.449 |  |
| SemSyn * Group effect | -0.01 | 0.22 | -0.04 | 0.966 |  |
| Control * Group effect | 0.11 | 0.15 | 0.74 | 0.462 |  |
| Syntactic * scale(Saliency) | 0.11 | 0.13 | 0.85 | 0.398 |  |
| Semantic * scale(Saliency) | -0.06 | 0.14 | -0.40 | 0.689 |  |
| SemSyn * scale(Saliency) | 0.02 | 0.17 | 0.09 | 0.930 |  |
| Control * scale(Saliency) | -0.11 | 0.10 | -1.09 | 0.276 |  |
| Group effect * scale(Saliency) | -0.03 | 0.13 | -0.26 | 0.796 |  |
| Syntactic * Group effect * scale(Saliency) | -0.11 | 0.17 | -0.66 | 0.508 |  |
| Semantic * Group effect * scale(Saliency) | -0.15 | 0.19 | -0.80 | 0.426 |  |
| SemSyn * Group effect * scale(Saliency) | -0.04 | 0.21 | -0.17 | 0.862 |  |
| Control * Group effect * scale(Saliency) | 0.09 | 0.14 | 0.63 | 0.529 |  |
|  |  |  |  |  |  |
| **First pass dwell time:** |  |  |  |  |  |
| **TLD is intercept** | *β* | *se* | *t* | *p* |  |
| (Intercept) | 5.80 | 0.10 | 56.15 | 0.000 | *** |
| Syntactic | 0.13 | 0.15 | 0.87 | 0.388 |  |
| Semantic | 0.18 | 0.15 | 1.23 | 0.221 |  |
| SemSyn | 0.34 | 0.16 | 2.08 | 0.041 | * |
| Control | 0.05 | 0.12 | 0.44 | 0.662 |  |
| Group effect | 0.11 | 0.14 | 0.77 | 0.440 |  |
| scale(Saliency) | 0.06 | 0.10 | 0.54 | 0.592 |  |
| Syntactic * Group effect | 0.02 | 0.20 | 0.12 | 0.907 |  |
| Semantic * Group effect | -0.16 | 0.21 | -0.76 | 0.449 |  |
| SemSyn * Group effect | 0.01 | 0.22 | 0.04 | 0.966 |  |
| Control * Group effect | -0.11 | 0.15 | -0.74 | 0.462 |  |
| Syntactic * scale(Saliency) | 0.00 | 0.13 | -0.02 | 0.985 |  |
| Semantic * scale(Saliency) | -0.20 | 0.14 | -1.44 | 0.152 |  |
| SemSyn * scale(Saliency) | -0.02 | 0.16 | -0.14 | 0.890 |  |
| Control * scale(Saliency) | -0.02 | 0.12 | -0.20 | 0.841 |  |
| Group effect * scale(Saliency) | 0.03 | 0.13 | 0.26 | 0.796 |  |
| Syntactic * Group effect * scale(Saliency) | 0.11 | 0.17 | 0.66 | 0.508 |  |
| Semantic * Group effect * scale(Saliency) | 0.15 | 0.19 | 0.80 | 0.426 |  |
| SemSyn * Group effect * scale(Saliency) | 0.04 | 0.21 | 0.17 | 0.862 |  |
| Control * Group effect * scale(Saliency) | -0.09 | 0.14 | -0.63 | 0.529 |  |
|  |  |  |  |  |  |
| **First fixation duration:** |  |  |  |  |  |
| **DLD is intercept** | *β* | *se* | *t* | *p* |  |
| (Intercept) | 5.73 | 0.07 | 83.82 | 0.000 | *** |
| Syntactic | -0.05 | 0.10 | -0.52 | 0.606 |  |
| Semantic | -0.07 | 0.10 | -0.73 | 0.467 |  |
| SemSyn | -0.06 | 0.11 | -0.55 | 0.585 |  |
| Control | -0.10 | 0.07 | -1.36 | 0.173 |  |
| Group effect | -0.13 | 0.10 | -1.38 | 0.169 |  |
| scale(Saliency) | -0.06 | 0.06 | -1.09 | 0.277 |  |
| Syntactic * Group effect | 0.01 | 0.14 | 0.06 | 0.955 |  |
| Semantic * Group effect | 0.11 | 0.13 | 0.85 | 0.399 |  |
| SemSyn * Group effect | 0.27 | 0.15 | 1.79 | 0.077 | # |
| Control * Group effect | 0.11 | 0.10 | 1.12 | 0.265 |  |
| Syntactic * scale(Saliency) | 0.10 | 0.08 | 1.31 | 0.193 |  |
| Semantic * scale(Saliency) | 0.05 | 0.09 | 0.54 | 0.588 |  |
| SemSyn * scale(Saliency) | -0.07 | 0.11 | -0.64 | 0.526 |  |
| Control * scale(Saliency) | 0.10 | 0.07 | 1.52 | 0.128 |  |
| Group effect * scale(Saliency) | 0.13 | 0.09 | 1.58 | 0.115 |  |
| Syntactic * Group effect * scale(Saliency) | -0.22 | 0.11 | -1.94 | 0.053 | # |
| Semantic * Group effect * scale(Saliency) | -0.06 | 0.13 | -0.48 | 0.632 |  |
| SemSyn * Group effect * scale(Saliency) | 0.00 | 0.15 | -0.01 | 0.996 |  |
| Control * Group effect * scale(Saliency) | -0.15 | 0.10 | -1.54 | 0.124 |  |
|  |  |  |  |  |  |
| **First fixation duration:** |  |  |  |  |  |
| **TLD is intercept** | *β* | *se* | *t* | *p* |  |
| (Intercept) | 5.60 | 0.07 | 82.20 | 0.000 | *** |
| Syntactic | -0.04 | 0.10 | -0.44 | 0.662 |  |
| Semantic | 0.04 | 0.09 | 0.46 | 0.644 |  |
| SemSyn | 0.21 | 0.10 | 2.00 | 0.049 | * |
| Control | 0.02 | 0.07 | 0.21 | 0.833 |  |
| Group effect | 0.13 | 0.10 | 1.38 | 0.170 |  |
| scale(Saliency) | 0.07 | 0.06 | 1.13 | 0.261 |  |
| Syntactic * Group effect | -0.01 | 0.14 | -0.05 | 0.958 |  |
| Semantic * Group effect | -0.11 | 0.13 | -0.85 | 0.396 |  |
| SemSyn * Group effect | -0.27 | 0.15 | -1.79 | 0.076 | # |
| Control * Group effect | -0.11 | 0.10 | -1.12 | 0.263 |  |
| Syntactic * scale(Saliency) | -0.12 | 0.08 | -1.43 | 0.155 |  |
| Semantic * scale(Saliency) | -0.01 | 0.09 | -0.12 | 0.905 |  |
| SemSyn * scale(Saliency) | -0.07 | 0.10 | -0.72 | 0.470 |  |
| Control * scale(Saliency) | -0.05 | 0.07 | -0.69 | 0.491 |  |
| Group effect * scale(Saliency) | -0.13 | 0.09 | -1.56 | 0.121 |  |
| Syntactic * Group effect * scale(Saliency) | 0.22 | 0.11 | 1.93 | 0.054 | # |
| Semantic * Group effect * scale(Saliency) | 0.06 | 0.13 | 0.46 | 0.649 |  |
| SemSyn * Group effect * scale(Saliency) | 0.00 | 0.15 | -0.02 | 0.983 |  |
| Control * Group effect * scale(Saliency) | 0.15 | 0.10 | 1.52 | 0.129 |  |
|  |  |  |  |  |  |
| **Saccade start time:** |  |  |  |  |  |
| **DLD is intercept** | *β* | *se* | *t* | *p* |  |
| (Intercept) | 2546.27 | 255.45 | 9.97 | 0.000 | *** |
| Syntactic | -131.27 | 347.08 | -0.38 | 0.706 |  |
| Semantic | -373.24 | 342.91 | -1.09 | 0.278 |  |
| SemSyn | -299.66 | 391.73 | -0.76 | 0.446 |  |
| Control | 308.74 | 296.24 | 1.04 | 0.299 |  |
| Group effect | 472.55 | 347.63 | 1.36 | 0.175 |  |
| scale(Saliency) | 38.80 | 202.13 | 0.19 | 0.848 |  |
| Syntactic * Group effect | -270.63 | 483.36 | -0.56 | 0.576 |  |
| Semantic * Group effect | -85.97 | 478.48 | -0.18 | 0.858 |  |
| SemSyn * Group effect | -466.35 | 492.36 | -0.95 | 0.344 |  |
| Control * Group effect | -551.21 | 392.45 | -1.40 | 0.161 |  |
| Syntactic * scale(Saliency) | 58.02 | 294.38 | 0.20 | 0.844 |  |
| Semantic * scale(Saliency) | 462.51 | 314.45 | 1.47 | 0.142 |  |
| SemSyn * scale(Saliency) | -126.41 | 446.34 | -0.28 | 0.778 |  |
| Control * scale(Saliency) | 149.40 | 258.73 | 0.58 | 0.564 |  |
| Group effect * scale(Saliency) | 359.43 | 306.11 | 1.17 | 0.241 |  |
| Syntactic * Group effect * scale(Saliency) | -1112.78 | 404.44 | -2.75 | 0.006 | ** |
| Semantic * Group effect * scale(Saliency) | -572.36 | 449.51 | -1.27 | 0.203 |  |
| SemSyn * Group effect * scale(Saliency) | -170.79 | 517.93 | -0.33 | 0.742 |  |
| Control * Group effect * scale(Saliency) | -306.30 | 351.19 | -0.87 | 0.384 |  |
|  |  |  |  |  |  |
| **Saccade start time:** |  |  |  |  |  |
| **TLD is intercept** | *β* | *se* | *t* | *p* |  |
| (Intercept) | 3019.57 | 261.92 | 11.53 | 0.000 | *** |
| Syntactic | -405.16 | 351.87 | -1.15 | 0.251 |  |
| Semantic | -459.12 | 331.20 | -1.39 | 0.168 |  |
| SemSyn | -766.13 | 374.42 | -2.05 | 0.045 | * |
| Control | -250.47 | 300.53 | -0.83 | 0.406 |  |
| Group effect | -470.88 | 345.80 | -1.36 | 0.174 |  |
| scale(Saliency) | 399.38 | 240.27 | 1.66 | 0.098 | # |
| Syntactic * Group effect | 273.69 | 483.55 | 0.57 | 0.572 |  |
| Semantic * Group effect | 90.93 | 479.25 | 0.19 | 0.850 |  |
| SemSyn * Group effect | 470.24 | 492.33 | 0.96 | 0.340 |  |
| Control * Group effect | 559.87 | 392.48 | 1.43 | 0.155 |  |
| Syntactic * scale(Saliency) | -1059.59 | 306.89 | -3.45 | 0.001 | *** |
| Semantic * scale(Saliency) | -111.29 | 329.27 | -0.34 | 0.736 |  |
| SemSyn * scale(Saliency) | -301.59 | 405.99 | -0.74 | 0.461 |  |
| Control * scale(Saliency) | -146.50 | 287.58 | -0.51 | 0.611 |  |
| Group effect * scale(Saliency) | -367.66 | 304.61 | -1.21 | 0.228 |  |
| Syntactic * Group effect * scale(Saliency) | 1115.72 | 401.81 | 2.78 | 0.006 | ** |
| Semantic * Group effect * scale(Saliency) | 576.88 | 448.39 | 1.29 | 0.199 |  |
| SemSyn * Group effect * scale(Saliency) | 199.78 | 513.35 | 0.39 | 0.697 |  |
| Control * Group effect * scale(Saliency) | 301.34 | 347.86 | 0.87 | 0.387 |  |

**Table S2.** Linguistic profile of children with DLD based on their CELF-4 performance.

|  | **Subtests** | **Number of children** |
| --- | --- | --- |
| Low performance at grammatical level only | Formulated Sentences | 3 |
|  | Formulated Sentences + Word Structure | 2 |
| Low performance at grammatical and semantic level | Formulated Sentences + Receptive Word Classes | 1 |
|  | Formulated Sentences + Expressive Word Classes | 2 |
|  | Formulated Sentences + Expressive Word Classes + Receptive Word Classes | 6 |
|  | Formulated Sentences + Word Structure + Receptive Word Classes | 1 |
|  | Formulated Sentences + Word Structure + WCE | 1 |
|  | Formulated Sentences + Word Structure + Expressive Word Classes + Receptive Word Classes | 3 |
|  | Word Structure + Expressive Word Classes + Receptive Word Classes | 1 |
